# A multi-centre evaluation of deep learning based radiotherapy planning for left-sided node-negative breast cancer

**DOI:** 10.1016/j.phro.2025.100839

**Published:** 2025-09-22

**Authors:** Marlie Besouw, Niels van Acht, Dave van Gruijthuijsen, Thérèse van Nunen, Jorien van der Leer, Maurice van der Sangen, Jacqueline Theuws, Jean-Paul Kleijnen, Antoinette Verbeek-de Kanter, Chrysi Papalazarou, Marcelle Immink, Roel Kierkels, Coen Hurkmans

**Affiliations:** a Radboudumc, Department of Medical Imaging, Nijmegen, the Netherlands; b Catharina Hospital, Department of Radiation Oncology, Eindhoven, the Netherlands; c Faculty of Electrical Engineering, Technical University Eindhoven, Eindhoven, the Netherlands; d Haaglanden Medical Centre, Department of Radiation Oncology, The Hague, the Netherlands; e Leiden University Medical Centre, Department of Radiation Oncology, Leiden, the Netherlands; f Radiotherapiegroep, Institution for Radiation Oncology, Arnhem, the Netherlands; g Faculty of Applied Physics, Technical University Eindhoven, Eindhoven, the Netherlands

**Keywords:** Breast cancer, Deep learning, Dose prediction, Dose mimicking, Multicenter validation, Radiotherapy planning

## Abstract

**Background and Purpose:**

Deep learning based planning (DLP) has the potential to improve consistency and efficiency in radiotherapy treatment planning. However, its clinical applicability remains limited, partly due to the need to translate a predicted dose into a deliverable dose. This study evaluated the generalisability of an institution specific DLP solution across multiple institutions by assessing its performance and developing a standardised translation parameter set.

**Materials and Methods:**

Four institutions provided clinical treatment plans of 15 patients with left-sided node-negative breast cancer. Treatment plans delivering 40.05 Gy were generated using a deep learning prediction model trained on data from one institution. External validation was performed using national consensus criteria, by applying the initial parameter settings (InitialMimick) to datasets (n = 45) from three other institutions. A standardised parameter set (GenericMimick) was then developed based on data (n = 12) from all four institutions, whereafter it was evaluated on the remaining 48 patients of the dataset.

**Results:**

InitialMimick plans showed higher average dose values in the planning target volume for the D_mean_ (40.5 vs. 40.1 Gy) and D_2%_ (42.4 vs. 41.4 Gy), with fewer cases meeting all clinical goals (15/45) compared to clinical plans (25/45). After parameter adjustment, GenericMimick plans resulted in more plans meeting all goals (28/48), comparable to the clinical plans (30/48), with D_mean_ of 40.3 vs. 40.1 Gy and D_2%_ of 41.9 vs. 41.5 Gy. Mean differences in organs at risk mean doses were less than 0.2 Gy.

**Conclusion:**

DLP with a standardised translation parameter set demonstrated general applicability across multiple institutions.

## Highlights

• Multicentre evaluation of deep learning based dose prediction for breast radiotherapy.
• Initial settings led to fewer plans meeting clinical goals than clinical planning.
• Optimised parameters were developed using patient data from four hospitals.
• Optimised plans met clinical goals at a level comparable to clinical planning.
• A standardised parameter set enables broader use of deep learning planning.

## Graphical abstract

**Figure:**
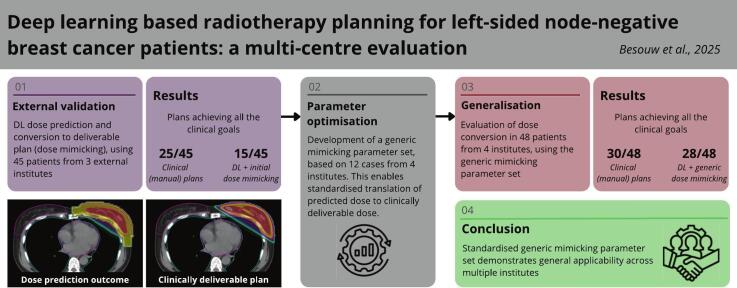

## 1 Introduction

Breast cancer is the most prevalent type of cancer and the leading cause of cancer-related mortality in women worldwide [1]. To improve local tumour control, radiotherapy is often included in treatment, particularly after breast-conserving surgery [2]. Radiotherapy planning is therefore a critical component of breast cancer care, as it impacts tumour control and the risk of radiation-induced side effects.

Conventionally, treatment plans are manually designed by medical physicists or radiotherapy technologists, involving beam arrangement selection, dose constraints, and optimisation parameters. However, this manual approach is time-consuming and prone to inter-planner variability, resulting in differences in treatment quality across institutions [3]. To address these challenges, deep learning (DL) based dose prediction models have been introduced [[4], [5], [6], [7]]. These models aim to predict patient-specific dose distributions based on delineations and CT planning data. Ahn et al. [4] developed a DL model for left-sided VMAT plans, demonstrating improved accuracy compared to a conventional knowledge-based planning approach. Hou et al. [5] proposed a 3D U‑Net model incorporating architectural enhancements for improved dose prediction in breast cancer, showing high accuracy and successful external validation. Both approaches from Ahn et al. and Hou et al. stopped at dose prediction and did not assess whether deliverable plans could be translated into clinically deliverable treatment plans. Implementation of such models may lead to significant time savings [8,9]. While DL based dose prediction may enhance efficiency, its raw output is not clinically deliverable and must be converted into a feasible plan through dose mimicking [10]. The mimicking algorithm translates the predicted dose distribution into a clinically feasible treatment plan by optimising treatment machine configurations and minimising deviations from the predicted dose. In this study, we refer to DL based planning (DLP) as the combined process of DL based dose prediction and mimicking.

The mimicking algorithm transforms dose predictions into clinically deliverable treatment plans by adjusting objective functions and their respective weights [11]. While these parameters are generally treatment protocol- and technique-specific, they are often fine-tuned at an institutional level rather than standardised [12]. This fine-tuning process is labour-intensive, relying on trial and error, making efficient implementation across different institutions challenging. Previously, a DL model for whole breast irradiation has been successfully implemented at a single institution with a site-specific mimicking parameter set [6]. This model utilises a 3D U-Net architecture and was trained on radiotherapy plans prescribing 40.05 Gy (15 fractions of 2.67 Gy). Previous work by Bakx et al. assessed its performance within this controlled setting [13]. However, the model’s ability to generalise across multiple institutions remained untested.

This study aimed to assess the performance of DLP across multiple institutions, with fine-tuning of the mimicking parameters based on a multi-institutional dataset. This generalised approach can help to consistently meet clinical criteria across institutions, thereby enhancing the feasibility of standardised deep learning driven treatment planning.

## 2 Materials and methods

The assessment of DLP performance was conducted in two stages. In the first stage, an external evaluation of the DLP model incorporating an institute-specific set of mimicking parameters, was carried out using a multi-institutional dataset. This step aimed to derive a standardised set of mimicking parameters applicable across diverse clinical settings, which was subsequently employed in the second stage of the evaluation. In the second stage, a standardised set of mimicking parameters was derived based on data from the original institution and external institutions combined.

### 2.1 Patient group

In this retrospective study, datasets were collected from four institutes, including the institute whose data was used to train the dose prediction algorithm. Ethical approval was not required, as the data were retrospective and anonymized. Each institute provided a dataset of 15 randomly selected patients diagnosed with left-sided node-negative breast cancer who had undergone a lumpectomy and sentinel lymph node biopsy followed by radiotherapy of the whole left breast. The radiotherapy treatment was administered in 15 daily fractions of 2.67 Gy, resulting in a cumulative dose of 40.05 Gy. All patients were treated in the breath-hold position with whole breast irradiation using intensity-modulated radiotherapy (IMRT), with a beam energy of 6 or 10 MV. Standard treatment protocols across these institutes involved the use of one lateral and one mediolateral beam. Treatment plan evaluations were performed according to the Dutch consensus criteria outlined by Hurkmans et al. [14].

The datasets include CT images with a spatial resolution of 512 x 512, a slice thickness of 3 mm, segmentations of the clinical target volume (CTV), heart, lungs and contralateral breast, and the clinical treatment plan including the corresponding dose generated by each institute. For this patient group, the breast planning target volume (PTV) is generated by expanding the CTV contour by 5 mm, and subsequently cropping the PTV to 5 mm beneath the skin.

There was no statistically significant difference in PTV volumes between the local (median 903 cm^3^ [IQR: 778–949 cm^3^]) and external cohorts (median 804 cm^3^ [IQR: 577–1121 cm^3^]) (p = 0.50). Both distributions were within the reported range of the original training population (196–2864 cm^3^) [6]. All treatment plans were developed specifically for each institute’s respective Elekta CBCT-equipped treatment machine and were manually optimised by experienced treatment planners.

### 2.2 Datasets for external validation

For the external validation, the performance of the DLP approach, was assessed on the three external datasets comprising a total of 45 patients. The DLP was applied using an initial mimicking parameter set specifically tailored for the local institute, hereafter referred to as InitialMimick. To ensure a fair comparison, the dataset from this institute was excluded from the external validation, as its inclusion could bias the results. The performance of the DLP on the local institute’s dataset is provided in Supplementary Table S1 and Fig. S1.

### 2.3 Datasets for standardised mimicking parameter set

By fine-tuning the InitialMimick parameter set through a trial and error process, a standardised set of mimicking parameters was derived, hereafter referred to as GenericMimick. These parameters can be found in Table S1. The optimisation process included 3 patients in the optimisation subset from each participating institute, including the local institute. The remaining 12 patients per institution comprised the test set, which was subsequently used to evaluate the performance of the GenericMimick configuration.

Characteristics of the dataset used for external validation, as well as the optimisation and test sets, including ROI volumes and doses, are summarised in Table 1. Statistical comparisons were performed using the Mann-Whitney U-test between the optimisation and test sets.

**Table 1:** Characteristics of the data split used for the external evaluation and the mimicking optimisation process. The comparison includes the volumes of the regions of interest (ROIs) and the corresponding doses of the clinical plans for the patients. The median and IQR is given for the volumes in cm^3^ and doses in Gy. Statistical differences between the optimisation and test sets were assessed using the two-sided Mann–Whitney U-test. All p-values > 0.05, indicating no statistically significant differences.

ROI	Characteristic		External evaluation (n = 45) *Institutes: 3*		Optimisation set (n = 12) *Institutes: 4*		Test set (n = 48) *Institutes: 4*		P-value Opt. vs test set
PTV	Volume	(cm^3^)	805	*[577–1121]*	738	*[469*–*919]*	878	*[624*–*1094]*	0.17
	Mean dose	(Gy)	40.1	*[40.0–40.2]*	40.1	*[39.8*–*40.1]*	40.1	*[40.1*–*40.2]*	0.12
	D_98%_	(Gy)	38.1	*[38.0–38.3]*	38.1	*[37.9*–*38.1]*	38.1	*[38.1*–*38.3]*	0.06
	D_2%_	(Gy)	41.4	[41.3–42.0]	41.4	*[41.3*–*41.6]*	41.5	*[41.3*–*41.7]*	0.50
Heart	Volume	(cm^3^)	649	*[607*–*685]*	682	*[638–752]*	647	*[614–686]*	0.28
	Mean dose	(Gy)	1.1	*[0.9*–*1.5]*	1.1	*[0.9–1.2]*	1.0	*[0.8–1.3]*	0.73
Lungs	Volume	(cm^3^)	4822	*[4098*–*5641]*	4509	*[3940–5411]*	4888	*[4171–5454]*	0.54
	Mean dose	(Gy)	2.8	*[2.1*–*2.9]*	2.2	*[1.8–3.0]*	2.3	*[2.0–2.6]*	0.66
Contralateral breast	Volume	(cm^3^)	735	*[555*–*1175]*	720	*[419–867]*	804	*[604–1180]*	0.15
	Mean dose	(Gy)	0.5	*[0.4*–*0.6]*	0.5	*[0.4–0.6]*	0.5	*[0.4–0.5]*	0.77

### 2.4 Plan generation

The delineations of the PTV and OARs were used to determine the two optimal beam angles for each patient. The software embedded in the treatment planning system (TPS) (RayStation version 12a) refines the beam angles through an optimisation process by improving PTV coverage and reducing dose to OARs, resulting in an offset from the initial gantry angles at 130 and 310 degrees. Bakx et al. explained this method previously in [15]. The DL model predicts a voxel-wise dose distribution, using binary masks of the PTV and OARs as an input. The predicted dose is independent from the beam configuration. After generating the dose prediction, the mimicking process is employed to render a clinically deliverable plan. The treatment plans were generated for each institute using their respective treatment machine configuration files.

### 2.5 Evaluation

Treatment plan evaluation included a comparison between the clinical plans and the plans generated using the DLP approach. The assessment was based on the number of plans that fulfilled all clinical goals, using the Dutch consensus criteria as the reference standard [14]. The PTV dose goals consist of the mean dose (D_mean_), the near-maximum dose (D_2%_), and the near-minimum dose (D_98%_). No dose normalisation technique was applied on the results.

This study evaluated the complete DLP, including the mimicking parameters, since the predicted dose is not clinically deliverable. The isolated performance of the DL model is reported in Supplementary Table S2.

As the objective is to minimise the dose to the OARs, and these doses naturally vary due to anatomical differences among patients, the consensus criteria do not define strict threshold values. In this study, two thresholds per organ, based on previous publications and clinical practice, were specified to evaluate the OARs D_mean_ dose goals [16,17]. The thresholds for the heart are 2 and 3 Gy. For the lungs, the thresholds were set to 3 and 6 Gy. Last, the contralateral breast were evaluated using the thresholds 1 and 2 Gy [18].

To assess statistical differences between clinical plans and plans generated with DLP, the two-sided Wilcoxon signed-rank test was applied (α = 0.05). Effect sizes were summarised as mean differences with 95 % confidence intervals (95 % CI), calculated using 5000 bootstrap samples per comparison. Confidence intervals reflect the percentile range of the bootstrap distribution.

## 3 Results

### 3.1 External evaluation

**Fig. 1:**
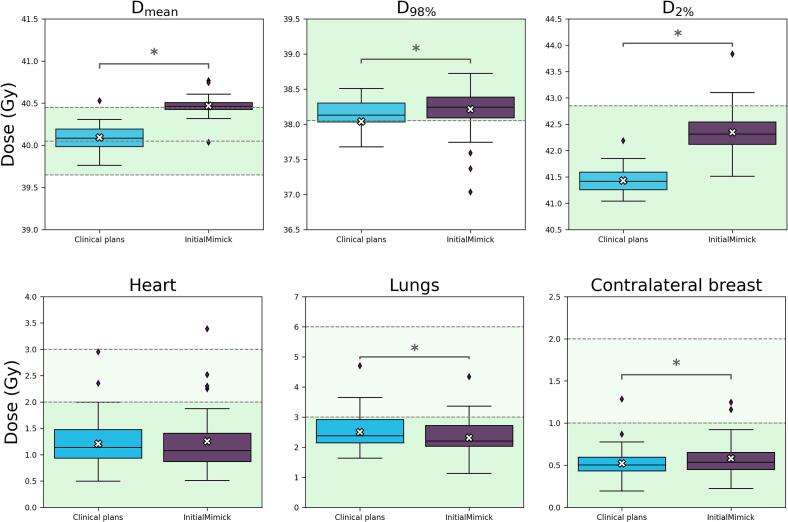
Boxplots of the results for the treatment plan evaluation metrics for the PTV and OARs across the multi-institutional dataset for the external evaluation (three institutes, n = 45). Boxplots show the median (horizontal line), interquartile range (box), and range (whiskers), with outliers shown as individual points. White crosses represent the mean dose per group. D_mean_: Mean dose, D_98%_: Minimum dose received by 98 % of the PTV volume, and D_2%_: Maximum dose received by 2 % of the PTV volume. The green shaded area denotes the threshold or acceptable dose range. The blue boxplots represent the clinical treatment plans generated by the institute, while the purple boxplots represent the results for the treatments plans generated using the DLP approach, using the initial mimicking parameters (InitialMimick). The asterisks indicate a statistically significant difference between the methods. (For interpretation of the references to colour in this figure legend, the reader is referred to the web version of this article.)

The InitialMimick plans resulted in statistically significant higher values for the PTV goals, with mean differences of 0.4 Gy (95 % CI: [0.3, 0.4 Gy]) for the D_mean_, 0.2 Gy (95 % CI: [0.0, 0.4 Gy]) for the D_98%_, and 0.9 Gy (95 % CI: [0.8, 1.0 Gy]) for D_2%_ (Fig. 1, Table 2). For the heart, no statistically significant dose difference was observed between the two planning approaches (p-value = 0.92). The mean dose difference to the lungs was 0.2  Gy lower in the InitialMimick plans (95 % CI: [–0.3, –0.1 Gy]), compared to the clinical plans. Conversely, the mean dose to the contralateral breast was 0.1  Gy higher (95 % CI: [0.0, 0.1 Gy]) in the InitialMimick plans than in the clinical plans.

**Table 2:** The median and IQR of all the treatment plans. For each method, 45 plans are evaluated. All dose values are displayed in Gy. Mean differences are calculated using 5000 bootstrap samples. 95% confidence intervals reflect the percentile range of the bootstrap distribution. Significance is calculated between the methods using the Wilcoxon signed rank test, where the asterisk denotes a significant difference between the methods’ outcomes.

Structure	Clinical goal [Gy]	Clinical plans (median *[IQR]* in Gy)		InitialMimick (median *[IQR]* in Gy)		Mean difference *[95 % CI] in Gy*	
PTV	39.65 ≤ D_mean_ ≤ 40.45	40.1	*[40.1–40.2]*	40.5	*[40.4–40.5]*	0.4*	*[0.3, 0.4]*
	D_98%_ ≥ 38.05	38.1	*[38.0–38.3]*	38.2	*[38.1–38.4]*	0.3*	*[0.0, 0.4]*
	D_2%_ ≤ 42.85	41.4	*[41.3–41.6]*	42.3	*[42.1–42.5]*	0.9*	*[0.8, 1.0]*
Heart	D_mean_	1.1	*[0.9–1.5]*	1.1	*[0.9–1.4]*	0.1	*[0.0, 0.1]*
Lungs	D_mean_	2.4	*[2.1–2.9]*	2.2	*[2.0–2.7]*	−0.2*	*[−0.3, −0.1]*
Contralateral breast	D_mean_	0.5	*[0.4–0.6]*	0.5	*[0.5–0.7]*	0.1*	*[0.0, 0.1]*

Among the 45 clinical plans, 25 met all PTV and OAR clinical goals when evaluated using the more stringent OARs criteria, compared to 15 out of 45 for the InitialMimick cases. When applying the less strict OARs criteria, these numbers increased to 32 and 18 cases, respectively. Considering only the PTV goals, these numbers remained unchanged, with 32 of the clinical plans and 18 of the InitialMimick cases that met all PTV criteria.

### 3.2 Alterations in the mimicking parameters

The external evaluation presented in Section 3.1 underscored the necessity of optimising the prediction and mimicking parameters to increase the proportion of plans that met all clinical goals, thereby aligning more closely with the performance of the clinical plans. Achieving this improvement required a reduction in both the D_mean_ and the D_2%_ to the PTV, while ensuring the D_98%_ was preserved. The alterations that had been made to improve the initial parameters can be found in Table 3. The complete set of prediction and mimicking parameters and its alterations can be found in the Supplementary Table S3.

**Table 3:** The alterations in the InitialMimick parameters leading to the GenericMimick parameter set.

Goal	Alteration
Decrease the D_mean_ and D_2%_ in the PTV	Decreased the dose of the maximum equivalent uniform dose (EUD) function
	Added maximum dose function on the PTV
Maintain the D_98%_	Increased minimum dose level on the PTV
	Increased the weight of the minimum dose level on the PTV
	Added a minimum EUD function

### 3.3 Plan results using the standardised mimicking parameter set

**Fig. 2:**
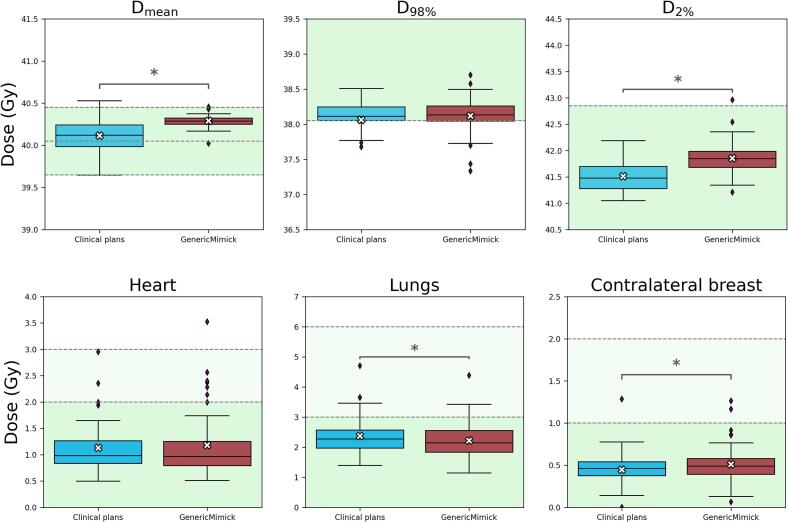
Boxplots of the results for the treatment plan evaluation metrics for the PTV and OARs across the multi-institutional dataset for the optimisation of the mimicking parameters (4 institutes n = 48). Boxplots show the median (horizontal line), interquartile range (box), and range (whiskers), with outliers shown as individual points. White crosses represent the mean dose per group. D_mean_: Mean dose, D_98%_: Minimum dose received by 98 % of the PTV volume, and D_2%_: Maximum dose received by 2 % of the PTV volume. The green shaded area represents the threshold or range of the acceptable dose. The blue boxplot represents the clinical treatment plans of the institute, and the red boxplots represent the results for the treatments plans generated using the DLP approach with generic mimicking parameters (GenericMimick). It should be noted that the clinical plan data in this analysis differ from those in the external validation, where the local institute’s data were excluded. The asterisk indicates a statistically significant difference between the methods. (For interpretation of the references to colour in this figure legend, the reader is referred to the web version of this article.)

For the PTV D_mean_, the clinical plans demonstrated greater variability than the GenericMimick plans. The latter showed a higher average dose, with a mean difference of 0.2 Gy (95 % CI: [0.1, 0.2 Gy]) (Fig. 2, Table 4). The difference between the methods for the D_98%_ goal was not considered statistically significant (p-value = 0.95). For D_2%_, the IQR was comparable between the clinical and the GenericMimick plans. However, the GenericMimick plans showed a higher average dose, with a mean difference of 0.3 Gy (95 % CI: [0.3, 0.3 Gy]). Only one outlier in the GenericMimick group exceeded the clinical threshold of 42.85 Gy.

**Table 4:** The median and IQR of all the treatment plans. For each method, 48 plans are evaluated. All dose values are displayed in Gy. Mean differences are calculated using 5000 bootstrap samples. 95 % confidence intervals reflect the percentile range of the bootstrap distribution. Statistical significance is determined using the two sided Wilcoxon signed rank test (α = 0.05), where asterisks indicate significant differences between methods.

Structure	Clinical goal [Gy]	Clinical plans (median *[IQR]*) in Gy		GenericMimick (median *[IQR]*) in Gy		Mean difference *[95 % CI]* in Gy	
PTV	39.65 ≤ D_mean_ ≤ 40.45	40.1	*[40.0–40.2]*	40.3	*[40.3–40.3]*	0.2*	*[0.1, 0.2]*
	D_98%_ ≥ 38.05	38.1	*[38.1–38.3]*	38.1	*[38.1–38.3]*	0.1	*[−0.1, 0.2]*
	D_2%_ ≤ 42.85	41.5	*[41.3–41.7]*	41.8	*[41.7–42.0]*	0.3*	*[0.3, 0.4]*
Heart	D_mean_	1.0	*[0.8–1.3]*	1.0	*[0.8–1.3]*	0.1	*[0.0, 0.1]*
Lungs	D_mean_	2.3	*[2.0–2.6]*	2.2	*[1.8–2.6]*	−0.2*	*[−0.2, −0.1]*
Contralateral breast	D_mean_	0.5	*[0.4–0.5]*	0.5	*[0.4–0.6]*	0.1*	*[0.0, 0.1]*

Regarding the OARs, the GenericMimick plans resulted in a 0.2 Gy reduction of the mean lung dose compared to the clinical plans (95 % CI: [0.1, 0.2 Gy]). Conversely, the mean dose to the contralateral breast was slightly higher by 0.1 Gy (95 % CI: [0.0, 0.1 Gy]). Consistent with findings from the external evaluation, no statistically significant difference was observed in heart dose (p = 0.75).

When evaluating the number of treatment plans that met all predefined PTV and OAR clinical goals, 30 out of 48 clinical plans fulfilled all stringent criteria, compared to 28 out of 48 for the GenericMimick plans. Under the more lenient thresholds (the lighter green region in Fig. 1), 37 out of 48 clinical plans and 34 out of 48 GenericMimick plans met the requirements. Considering only the PTV-related goals, 37 out of 48 clinical plans and 35 out of 48 GenericMimick plans achieved all associated criteria.

## 4 Discussion

The most notable finding of this study was that using a multi-institutional optimised mimicking parameter set, enabled plan quality comparable to clinical plans across multiple institutions. Differences in OARs doses remained clinically insignificant. As such, these settings could be used as a good starting point for creating clinical plans. For a minority of plans, manual fine-tuning may still be considered, although prior work has suggested that in some cases, this does not lead to improved outcomes.

These findings align with previous work. Borderías-Villaroel et al. [18] highlighted that one of the key challenges in generalising DLP lies in the post-processing stage, particularly in the definition of mimicking parameters. Other studies have similarly reported clinically insignificant OAR differences [19] and limited benefit of manual fine-tuning [13]. Rather than retraining the model, which is computationally and labour-intensive, this study focused on optimising the mimicking parameters step to improve clinical goal fulfilment. While most published work on DL dose mimicking focuses on proton therapy settings [[20], [21], [22]], our study contributes to the growing body of evidence supporting its use in photon-based techniques. Although not based on DL, Babier et al. [23] proposed an automated evaluation framework based on dose score to compare plans. Due to weak correlation between this metric and the clinical plan ranking, it was not used in this study.

A major limitation of this study was the manual adjustment of mimicking parameters through trial and error, making it infeasible to optimise for larger datasets within the current software framework. This approach introduces subjectivity and increases the risk of suboptimal parameter selection, as small adjustments can lead to substantial outcome changes. Moreover, some functions in the mimicking algorithm are interdependent, resulting in either opposing or synergistic effects. Additionally, this study was conducted across four institutes using similar radiotherapy techniques (IMRT), limiting applicability to institutions with different planning techniques or treatment machines. Zeverino et al. showed the feasibility of applying a VMAT-based model for left-sided breast cancer with a simultaneous integrated boost to right-sided casing using a similar approach, without retraining [24].

The mimicking parameters were optimised based on data from all three external institutes. To demonstrate generalisability, an independent external dataset would be required. To overcome current limitations, future work should prioritise automated generation of mimicking parameters tailored to specific patient groups. This would enhance scalability, support broader clinical adoption across institutions with varying techniques and equipment, and facilitate the extension of DLP to, for example, locoregional breast radiotherapy.

Initial prospective work in other indications, such as prostate cancer, has already demonstrated the clinical acceptability and efficiency gains of AI-based treatment planning [25], further supporting the relevance of such evaluations in photon-based DLP applications. Ultimately, validation through prospective, multi-institutional studies will be essential to confirm clinical feasibility and robustness.

In conclusion, this study demonstrates the general applicability of deep learning based whole breast radiotherapy planning among four institutes, achieving results comparable to clinical treatment plans when using a mimicking parameter set optimised with multi-institutional data. Manual parameter fine-tuning remains labour intensive, underscoring the need for automated parameter optimisation techniques.

## CRediT authorship contribution statement

**Marlie Besouw:** Methodology, Formal analysis, Investigation, Data curation, Writing – original draft, Visualization. **Niels van Acht:** Methodology, Resources, Writing – review & editing. **Dave van Gruijthuijsen:** Methodology, Resources, Writing – review & editing. **Thérèse van Nunen:** Resources. **Jorien van der Leer:** Resources, Writing – review & editing. **Maurice van der Sangen:** Resources, Writing – review & editing. **Jacqueline Theuws:** Resources. **Jean-Paul Kleijnen:** Resources, Writing – review & editing. **Antoinette Verbeek-de Kanter:** Resources. **Chrysi Papalazarou:** Resources, Writing – review & editing. **Marcelle Immink:** Resources, Writing – review & editing. **Roel Kierkels:** Resources, Writing – review & editing. **Coen Hurkmans:** Conceptualization, Methodology, Writing – review & editing, Supervision.

## Declaration of competing interest

The authors declare the following financial interests/personal relationships which may be considered as potential competing interests: Niels van Acht received funding from RaySearch Laboratories AB. RaySearch Laboratories AB had no influence on the design or reporting of the study.
