# Supplementary material for: A multi-centre evaluation of deep learning based radiotherapy planning for left-sided node-negative breast cancer
Source: Phys Imaging Radiat Oncol. 2025 Sep 22;36:100839. doi: 10.1016/j.phro.2025.100839 (PMC12492285; doi:10.1016/j.phro.2025.100839)
Supplement: Supplementary Data 1 [file mmc1.pdf]

APPENDIX

Supplementary Table S1:

The median and IQR of the treatment plans for the local institute. For each method, 15 plans are evaluated. All dose values are displayed in Gy. Mean differences are calculated using 5000 bootstrap samples. 95% confidence intervals reflect the percentile range of the bootstrap distribution. Significance is calculated between the methods using the Wilcoxon signed rank test, where the asterisk denotes a significant difference between the methods' outcomes. For the clinical plans, 12 of 15 plans achieved all goals, decreasing to 11 of 15 when evaluating the more stringent goals. For the DLP approach using InitialMimick, this number was 9 out of 15 plans and remained the same when evaluating the more stringent goals.

Structure	Clinical goal [Gy]	Clinical plans: Local institute (n=15) (median [IQR] in Gy)		InitialMimick: Local institute (n=15) (median [IQR] in Gy)		Mean difference *[95% CI]* in Gy	
PTV	$39.65 \leq D_{mean} \leq 40.45$	40.2	*[39.8–40.3]*	40.4	*[40.4–40.5]*	0.3*	*[0.2, 0.5]*
	$D_{98\%} \geq 38.05$	38.1	*[38.1–38.1]*	38.2	*[38.1–38.4]*	0.2*	*[0.1, 0.3]*
	$D_{2\%} \leq 42.85$	41.8	*[41.6–41.9]*	42.5	*[42.2–42.7]*	0.7*	*[0.6, 0.9]*
Heart	D_{mean}	0.8	*[0.7–1.0]*	0.8	*[0.7–1.0]*	0.0	*[0.0, 0.0]*
Lungs	D_{mean}	1.8	*[1.5–2.1]*	1.7	*[1.4–2.0]*	-0.1	*[-0.2, 0.0]*
Contralateral breast	D_{mean}	0.3	*[0.1–0.4]*	0.3	*[0.2–0.4]*	0.1*	*[0.0, 0.1]*

Supplementary Table S2:

The median and IQR of the predicted dose for the local institute (n=15) and the 3 external institutes (n=45). All dose values are given in Gy. For the local institute, all 15 patients met all dose goals when evaluating the predicted doses. For the external institutes, 45 out of 45 patients met the goals. When applying the more stringent OAR goals, this number decreased to 34 out of 45.

Structure	Clinical goal [Gy]	Predicted dose: Local institute (n=15) (median *[IQR]* Gy)		Predicted dose: External institutes (n=45) (median *[IQR]* in Gy)	
PTV	$39.65 \leq D_{mean} \leq 40.45$	40.0	*[40.0–40.0]*	40.0	*[40.0–40.0]*
	$D_{98\%} \geq 38.05$	38.8	*[38.8–38.8]*	38.8	*[38.8–39.0]*
	$D_{2\%} \leq 42.85$	40.3	*[40.3–40.3]*	40.3	*[40.3–40.3]*
Heart	D_{mean}	0.9	*[0.8–1.2]*	1.3	*[0.9–1.7]*
Lungs	D_{mean}	1.9	*[1.6–2.2]*	2.4	*[2.1–2.8]*
Contralateral breast	D_{mean}	0.4	*[0.3–0.6]*	0.5	*[0.4–0.6]*

Supplementary Table S3:

The parameter configuration for the mimicking algorithm: The bold text represents the alterations made to configure the GenericMimick parameters. The strikethrough text denotes the value of the InitialMimick settings.

PredictSettings – RoiGoals

Goal Type	Function	Dose Level (cGy)	Volume Threshold	ROI
MaxDose	WeightedDvhShift	3955	0.5	PTV
MinDose	DvhShift	3955	0.5	PTV
MinDose	Rescale	(~~3795~~) **3895**	0.98	PTV
MinDose	Aggressive	3815		PTVptoSkin
MinDose	Aggressive	3799		PTVWall5mm-PTV
MinDose	Aggressive	(~~3839~~) **3849**		BeamArea

MimicSettings – Functions

ROI	Function	Weight	Additional Parameters
PTV	MinRefDose	(~~25~~) **35**	
PTV	MaxRefDose	1	IsoWeights: [1, 1, 1, 1, 1]; IsoDose: [106, 102, 100, 95, 0]
Heart	MaxRefDose	1	
Lungs	MaxRefDose	1	
ExternalWall-PTVptoSkin	MaxRefDose	3.0	
External	MaxExternalRefDose	5	MaxDoseThreshold: 3972.75; TargetRois: [PTV, PTVptoSkin]

MimicSettings – RoiGoals

ROI	Goal Type	Weight	Weight Scale Per Run	Value (cGy)
External-PTVptoSkin	MaxDose	2	[0, 0.5, 1, 1]	4156

RayStationObjectiveFunctions

ROI	Function	Weight	Dose Level (cGy)	Volume	EUD A	Apply Before Run Index
External	MaxDose	10000	4156			0
PTV	MaxDvh	10000	4203	0.01		1
PTV	MaxEud	10000	(~~4020~~) **4000**		1	2
PTV	MinDvh	15000	3855	0.99		2
**PTV**	**MinEud**	**10000**	**3900**		**1**	**2**
**PTV**	**MaxDose**	**10000**	**4100**			**0**

Supplementary Figure S1:

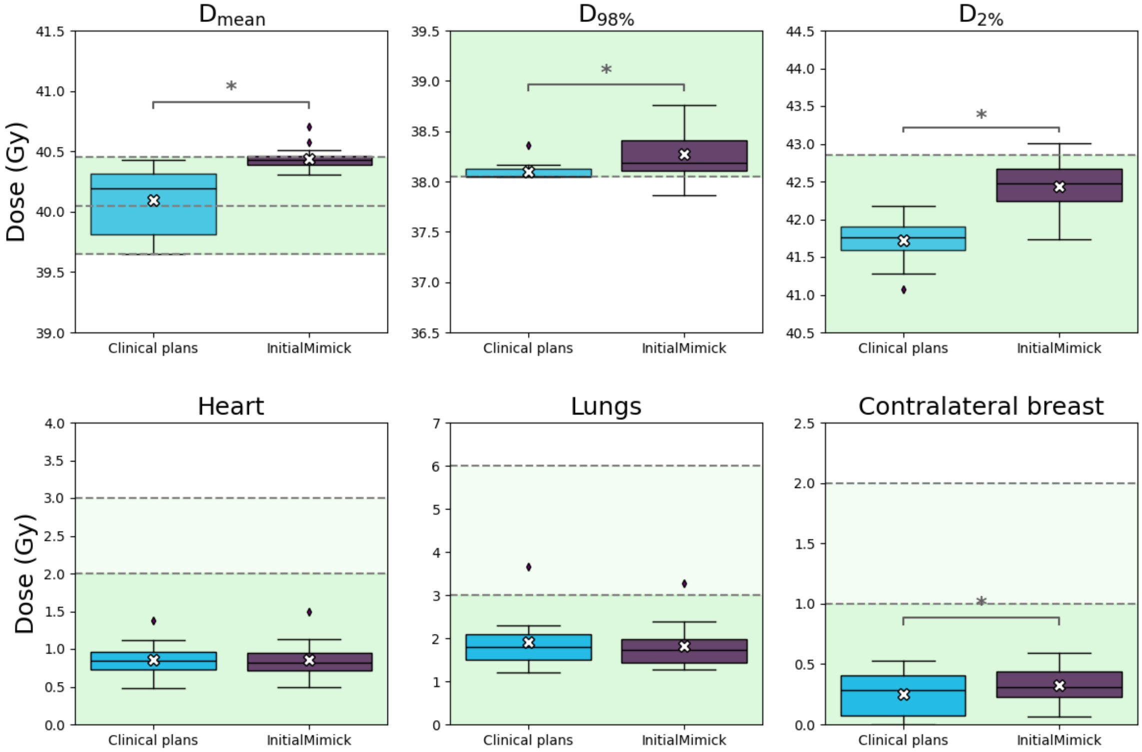

Boxplots of the results for the treatment plan evaluation metrics for the PTV and OARs dataset for the local institute's dataset (n=15). Boxplots show the median (horizontal line), interquartile range (box), and range (whiskers), with outliers shown as individual points. White crosses represent the mean dose per group. D_{mean}: Mean dose, $D_{98\%}$: Minimum dose received by 98% of the PTV volume, and $D_{2\%}$: Maximum dose received by 2% of the PTV volume. The green shaded area denotes the threshold or acceptable dose range. The blue boxplots represent the clinical treatment plans generated by the institute, while the purple boxplots represent the results for the treatments plans generated using the DLP approach, using the initial mimicking parameters (InitialMimick). The asterisks indicate a statistically significant difference between the methods.
